# Validation of the Greek version of Mothers on Respect (MOR) index

**DOI:** 10.18332/ejm/196694

**Published:** 2025-01-15

**Authors:** Eleni Serpetini, Antigoni Sarantaki, Aikaterini Lykeridou, Maria Vlachou, Athina Diamanti

**Affiliations:** 1 Department of Midwifery, School of Health and Care Sciences, University of West Attica, Athens, Greece

**Keywords:** mothers on respect index, maternity, pregnancy, home birth, childbirth, midwifery, autonomy

## Abstract

**INTRODUCTION:**

Pregnancy is a critical period marked by vast changes, with a pivotal role in healthcare. The Mothers on Respect (MOR) index measures and ensures respect in maternal care, impacting health-seeking behaviors and postpartum outcomes vital for individual and healthcare system well-being. This study aims to validate the Greek version of the MOR index to enhance respectful maternity care and contribute to positive childbirth experiences.

**METHODS:**

A retrospective, cross-sectional, descriptive, and analytical online survey collected data from Greek women with home childbirth experience. We utilized a self-administered questionnaire and the Greek version of the Mothers on Respect (MOR) index.

**RESULTS:**

The MOR index, assessing knowledge and awareness, showed a median score of 78 points, with a significant association between higher scores and living in Attica or being a healthcare professional (p=0.027 and p=0.024, respectively). Confirmatory factor analysis indicated the questionnaire had an acceptable fit, and reliability was confirmed with Cronbach’s α exceeding 0.7 across all dimensions.

**CONCLUSIONS:**

The Greek MOR index validation advances respectful maternity care, enhances maternal health in Greece, and contributes to regional efforts for positive childbirth experiences.

## INTRODUCTION

In contemporary society, the pregnancy journey represents a key period in a woman’s life, heralding significant physical, emotional, and psychological changes. Respect forms the cornerstone of patient-centered care, serving as a fundamental human right within healthcare frameworks globally^1^. During pregnancy, the importance of respect amplifies as women navigate a multitude of physical discomforts, emotional fluctuations, and existential concerns^2^. Research underscores the profound impact of respectful maternity care on maternal health-seeking behaviors, birth experiences, and postpartum outcomes^3,4^. Therefore, developing tools to measure and uphold respect within the realm of maternal healthcare becomes imperative, with implications for both individual well-being and broader healthcare systems^5^.

The Mothers on Respect (MOR) index emerges as a vital instrument in assessing and ensuring the fulfillment of respect for pregnant women within healthcare settings. The MOR index, conceived as a comprehensive measure of respect in maternal healthcare, encompasses various dimensions crucial to the experiences of pregnant women. Rooted in dignity, autonomy, and person-centered care principles, this index comprises multifaceted domains, including communication, decision-making autonomy, privacy, and emotional support^6^. Its development stems from a growing recognition of the need for standardized, evidence-based approaches to assess and enhance the quality of maternity care worldwide^7^. Previous validation studies in diverse cultural settings have demonstrated promising results, indicating its adaptability and utility across healthcare contexts^8,9^.

Culture influences health beliefs, practices, and expectations, shaping the dynamics of healthcare interactions and outcomes^10^. Pregnancy holds deep cultural significance within the Greek context, intertwining notions of family, tradition, and spirituality. Women in Greece may prioritize familial involvement, seek holistic approaches to care, and place significant trust in healthcare providers. Moreover, historical experiences and societal norms construct maternal identities and expectations surrounding childbirth^11^. Understanding these cultural nuances is essential in tailoring healthcare interventions and assessing the applicability of existing tools such as the MOR index.

The majority of Greek women choose to give birth in hospitals or maternity clinics, where they can access medical professionals and necessary equipment for safe deliveries^12^. However, a small percentage (0.29%) opt for home births, often seeking a more personalized and natural birthing experience. Certified midwives support home births in Greece, but these are primarily facilitated through private arrangements rather than those provided by the Ministry of Health and Primary Healthcare. Midwifery-led care is available and increasingly recognized for its benefits, particularly within hospital settings and private birthing centers^12,13^.

This study aims to validate the MOR index in women with at least one home childbirth in Greece. Specific objectives include assessing the psychometric properties of the index, exploring cultural adaptations and nuances, and examining its association with demographic characteristics.

## METHODS

### Study design and setting

Using an online self-administered questionnaire, we conducted a retrospective and cross-sectional, descriptive, and analytical online survey among women with at least one home childbirth experience in Greece from January 2010 to December 2023.

### Participants

The data collection sample consisted of Greek women with at least one home childbirth experience. The inclusion criteria were: 1) aged>18 years; and 2) experienced their last pregnancy within ten years of data collection. Responses about childbirth experiences from outside Greece were excluded from the dataset.

The study employed a non-probability sampling method, specifically convenience sampling. This method was selected due to the accessibility and willingness of participants in online groups dedicated to home childbirth. Women with homebirth experiences were chosen as participants for the validation of the MOR index because they represent a unique group with distinct perspectives on respectful maternity care. Homebirths are often associated with higher levels of autonomy, personalized care, and awareness of childbirth rights, which are central to the MOR index.

We developed a self-administered anonymized questionnaire using the Microsoft Forms electronic platform. The URL link to this questionnaire was shared on social media (Facebook) in closed online groups involving women who have given birth at home and engaging in group discussions on parenthood. A special infographic was created to attract women who have given birth at home, and sent electronically to women’s groups, midwife organizations, and associations that have given birth at home. Participation in the survey was voluntary, and prior to commencing the questionnaire, a concise paragraph was presented to inform participants about the study’s objectives and to guarantee the confidentiality of their responses.

### Measurements

*Instrument and scale*

The authors designed a self-administered questionnaire for the research needs to collect data on demographic characteristics, childbirth preparation, professional attendance during home birth, and knowledge of childbirth rights. The questionnaire comprised 11 questions divided into three sections (Supplementary file):

1. Demographics: Questions 1 to 6 covered age, nationality, place of residence, education level, marital status, and number of children.
2. Preparation and Professional Attendance: Questions 7 to 10 gathered information about antenatal preparation courses, household income, occupation, and the professionals who attended the home birth.
3. Knowledge of Childbirth Rights: Question 11 assessed participants’ awareness of hospitalized patients’ rights, home birth laws, children’s rights, and sexual and reproductive rights.

Additionally, the Greek version of the MOR index was utilized. This index, originally developed to measure respect in maternal healthcare, includes 14 items rated on a 6-point Likert scale. The scoring ranges from 1 (strongly disagree) to 6 (strongly agree) for most items, with some items reverse-scored. The total score ranges from 14 to 84 points, with higher scores indicating more respectful care^6^.

The instrument was pilot-tested to check the clarity of the questions and identify any features that might need modification, then translated and back translated to ensure accuracy.

### Ethical considerations

The study protocol was approved by the Research Ethics Boards of the University of West Attica (protocol number 77034/01-09-2023). All participants provided informed consent, and data collection was conducted anonymously. Participants were informed about the study’s objectives, and their responses were kept confidential to ensure their privacy and compliance with ethical standards.

### Statistical analysis

The distributions of the quantitative variables were tested for normality using the Kolmogorov-Smirnov test. Mean values and standard deviations (SD) were used for normally distributed variables, while medians and ranges were used for non-normally distributed variables. Absolute and relative frequencies described qualitative variables. The non-parametric Mann-Whitney U and Kruskal-Wallis tests were employed to compare non-normally distributed variables among different categories. Confirmatory factor analysis (CFA) was conducted using a maximum likelihood procedure to test the construct validity and confirm the factors of the MOR index. Fit indices such as the comparative fit index (CFI), Tucker-Lewis index (TLI), and root mean square error of approximation (RMSEA) were used to assess the model fit. The reliability of the questionnaire was confirmed with Cronbach’s α, which exceeded 0.7 across all dimensions, indicating acceptable reliability. The statistical analysis was performed using the statistical program IBM SPSS Version 26.0.

## RESULTS

### Sociodemographic and maternity history characteristics

Approximately 200 women were identified as potentially eligible based on their membership in relevant online groups and organizations related to home childbirth in Greece. Of these, 180 women accessed the online survey. One hundred sixty-two women met the inclusion criteria (aged >18 years, agreement to participate, and last pregnancy within the last ten years). All 162 eligible participants completed the survey. Data from all 162 participants were included in the final analysis. Reasons for non-participation at various stages included not meeting the inclusion criteria (e.g. childbirth experiences outside Greece). There were no significant missing data in the responses. All 162 participants completed the questionnaire, ensuring a 100% response rate for all questions. The response rate for the participants was 90%, calculated from the number of women who accessed the survey (180) and the number who completed it (162).

The final participants had a mean age of 36.4 years (SD=5.4 years); 94.4% of the participants had Greek nationality, and 54% lived in Attica. Also, 50.6% had a Bachelor’s degree, 82.1% were married, and 46.9% had two children. Table 1 summarizes the sociodemographic and maternity history characteristics of the study population.

**Table 1:** Sociodemographic characteristics of the study population

*Characteristics*	*Categories*	*n*	*%*
**Age** (years), mean (SD)		36.4 (5.4)	
**Nationality**	Other	9	5.6
	Greek	153	94.4
**Prefecture of residence: Attica**	No	74	46
	Yes	87	54
**Education level**	No education	1	0.6
	High school	7	4.3
	Technical school	3	1.9
	Vocational school for two years of training	13	8
	Bachelor’s degree	95	50.6
	Master’s degree	51	31.5
	Doctorate	5	3.1
**Marital status**	Married	133	82.1
	Single	5	3.1
	Divorced	4	2.5
	Cohabitation agreement	15	9.3
	Cohabitation	5	3.1
**Number of children**	1	28	17.3
	2	76	46.9
	3	42	25.9
	4	8	4.9
	5	5	3.1
	6	3	1.9

### Preparation for home birth, data regarding income and profession before the birth, and data on which professionals attended the home birth

The majority of participants (71%) attended antenatal preparation classes by a midwife before delivery. Half of them (46.3%) had a monthly family income of 1000–2000 €, and most (34.2%) were private employees; 21.7% were self-employed, 13.7% unemployed and 10.6% civil servants. Among the participants, 73.5% had two midwives during the home birth, and 13% had only a doula. Data regarding preparation for home birth, data regarding income and profession before the birth, and data on which professionals attended the home birth are illustrated in Table 2.

**Table 2:** Data regarding preparation for home birth, income and profession before the birth, and which professionals attended the home birth

*Questions*	*Categories*	*n*	*%*
**In your first home birth, did you attend antenatal preparation courses with a midwife?**	No	38	23.5
	Yes	115	71
	I am a midwife	9	5.6
**Monthly household income (€) during your first home birth?**	500–1000	46	28.4
	1001–2000	75	46.3
	2001–3000	26	16
	3001–4000	8	4.9
	>4000	7	4.3
**What was your job when you had your first home birth?**	Private employee	55	34.2
	Civil servant	17	10.6
	Self-employed	35	21.7
	Healthcare professional	8	4.9
	Midwife	9	5
	Unemployed	22	13.7
	Householder	16	9.9
**Which professionals attended your first home birth?**	2 midwives	119	73.5
	1 midwife	17	10.3
	1 midwife and 1 doula	11	6.8
	1 midwife and 1 gynecologist	5	3.1
	1 doula	21	13
	Gynecologist	6	3.7
	Pediatrician	5	3.1
	Acupuncturist	3	1.9
	Reflexologist	2	1.2
	Osteopath	1	0.6
	Unassisted	4	2.5
	Other	10	6.2

### Knowledge about childbirth rights and laws

Among the participants, 32.1% were very to extremely aware of hospitalized patient’s rights, 39.5% of current home birth laws, 43.8% of children’s rights, and 48.8% of sexual and reproductive rights. Table 3 illustrates the data regarding the knowledge about childbirth rights and laws.

**Table 3:** Knowledge about childbirth rights and laws

*To what extent do you know*	*Categories*	*n*	*%*	*Percent response very well - extremely well*
**The rights of hospitalized patient?**	Not at all	16	9.9	**32.1**
	Slightly	56	34.6	
	Moderately	38	23.5	
	Very well	22	13.6	
	Extremely well	30	18.5	
**The current home birth laws?**	Not at all	7	4.3	**39.5**
	Slightly	42	25.9	
	Moderately	49	30.2	
	Very well	31	19.1	
	Extremely well	33	20.4	
**The children’s rights?**	Not at all	8	4.9	**43.8**
	Slightly	30	18.5	
	Moderately	53	32.7	
	Very well	36	22.2	
	Extremely well	35	21.6	
**Your sexual and reproductive rights?**	Not at all	10	6.2	**48.8**
	Slightly	25	15.4	
	Moderately	48	29.6	
	Very well	38	23.5	
	Extremely well	41	25.3	

### The MOR index

The MOR index ranged from 16 to 84 points, with a median value of 78 points.

Table 4 illustrates MOR index item score percentages according to participants’ answers. From the MOR index responses, the following key findings were reported about decision-making regarding pregnancy and childbirth care.

**Table 4:** MOR index item score (%) according to participants’ answers

*Items*	*Strongly disagree*	*Disagree*	*Somewhat disagree*	*Somewhat agree*	*Agree*	*Strongly agree*
**Overall while making decisions about my pregnancy or birth care**						
I felt comfortable asking questions	1	1	2	8	15.7	71.6
I felt comfortable declining care that was offered	2.9	5.9	9.8	12.7	35.3	33.3
I felt comfortable accepting the options for care that my doctor or midwife recommended	0.6	2.5	3.7	9.3	25.9	58
I felt pushed into accepting the options my doctor or midwife suggested	63	19.8	3.7	6.2	4.3	3.1
I chose the care options that I received	1	2.9	4.9	13.7	20.6	56.9
My personal preferences were respected	0	1	2.9	8.8	12.7	74.5
My cultural preferences were respected	2	0	1	7.8	10.8	78.4
**Reasons why I felt that I was treated poorly by my doctor or midwife during my pregnancy**						
My race, ethnicity, cultural background or language	3.9	0	1	2.9	4.9	87.3
My sexual orientation and/or gender identity	4.9	0	0	2	5.9	87.3
My type of health insurance or lack of insurance	4.9	0	0	2	7.8	85.3
A difference of opinion with my caregivers about the right care for myself or my baby	5.9	2.9	2	1	6.9	81.4
**Reasons why I held back from asking questions or discussing my concerns during my pregnancy**						
My doctor or midwife seemed rushed	2.5	2.5	5.6	3.1	14.2	72.2
I wanted maternity care that differed from what my doctor or midwife recommended	3.7	2.5	4.9	3.1	9.9	75.9
I thought my doctor or midwife might think I was being difficult	1.9	3.7	4.9	6.2	13.6	69.8

### Confirmatory factor analysis

Confirmatory factor analysis revealed an acceptable fit for the questionnaire, where the CFI and TLI indices were 0.87 and 0.83, respectively, and the RMSEA index was acceptable and equal to 0.07.

The correlation coefficients of each question with the overall respect dimension in decision-making regarding pregnancy or childbirth care, the dimension regarding the behavior of a doctor or midwife during pregnancy, and the dimension regarding the possibility of asking questions and discussing concerns with the doctor or midwife during pregnancy were acceptable, as shown in Table 5. Also, it would not improve the reliability factor if any of the questions were removed, so all questions remain within the factor. The correlation coefficients in Table 5 were measured using Pearson’s r, which ranges from -1 to 1. Cronbach’s α reliability coefficient was greater than 0.7 for all dimensions, indicating acceptable reliability. Table 5 presents the correlations of the questions and the Cronbach’s reliability coefficient.

**Table 5:** Correlations of the questions and Cronbach’s reliability coefficient

*Dimension*	*Corrected item-total correlation*	*Cronbach’s alpha if item deleted*	*Cronbach’s alpha*
**Dimension about respect in making decisions about my pregnancy or childbirth care**			
MOR-1	0.510	0.814	**0.83**
MOR-2	0.503	0.821	
MOR-3	0.682	0.788	
MOR-4	0.431	0.835	
MOR-5	0.591	0.801	
MOR-6	0.801	0.777	
MOR-7	0.661	0.793	
**Dimension regarding the behavior of a doctor or midwife during pregnancy**			
MOR-8	0.835	0.922	**0.92**
MOR-9	0.952	0.918	
MOR-10	0.961	0.915	
MOR-11	0.830	0.934	
**Dimension regarding the possibility of asking questions and discussing concerns with the doctor or midwife during pregnancy**			
MOR-12	0.749	0.774	**0.85**
MOR-13	0.762	0.763	
MOR-14	0.670	0.847	

* MOR: Mothers on Respect.

### Association of MOR index score with participants’ characteristics

**Figure 1:**
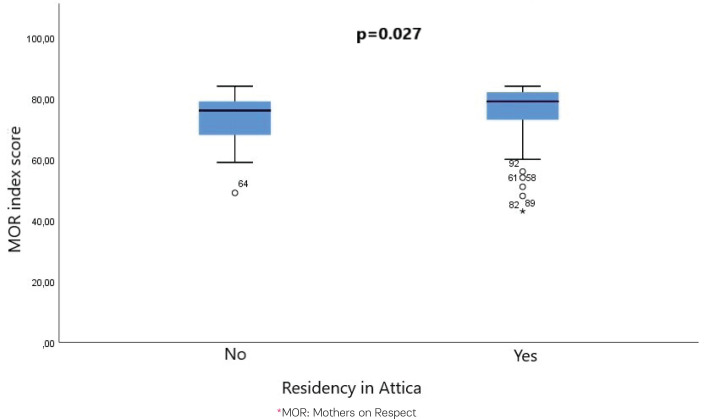
Median values of MOR index score in the women who lived in Attica during the first home birth compared to those who lived in other places, January 2010 to December 2023, Greece (N=162)

**Figure 2:**
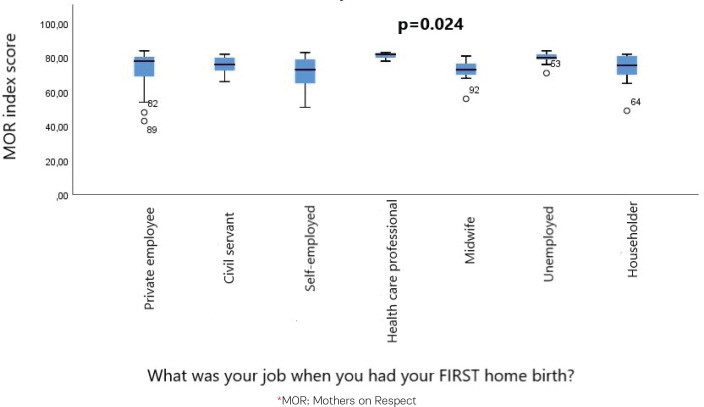
Median values of MOR index score among women with different professions, January 2010 to December 2023, Greece (N=162)

The study showed a statistically significant higher median value of MOR index score in the women who lived in Attica during their first home birth compared to those who lived in other places [MOR index score 79 (range: 49–84) vs 76 (range: 43–84); p=0.027, Mann-Whitney U test] (Figure 1). The study showed a statistically significant difference in the median values of MOR index scores among women in different professions, with the higher median value being detected among healthcare professionals (p=0.024, Kruskal Wallis test and Dunn’s test for *post hoc* pairwise comparison) (Figure 2).

## DISCUSSION

The present study validated the Greek version of the MOR index, providing a reliable tool for assessing respectful maternity care. Key findings include significant associations between higher MOR index scores and participants living in Attica or being healthcare professionals. The MOR index demonstrated a higher median value among these groups, suggesting regional and occupational differences in maternity care experiences. The study also highlighted the importance of decision-making autonomy, respectful treatment by healthcare professionals, and open communication during pregnancy and childbirth.

The validation of the Greek version of the MOR index is a pivotal advancement in understanding the experiences of women during pregnancy and childbirth within the Greek context. The psychometric properties of the MOR index demonstrate its reliability and validity as a comprehensive assessment tool for evaluating women’s experiences of respect during pregnancy and childbirth. This aligns with previous research, highlighting the importance of assessing respectful maternity care using this tool to improve maternal health outcomes^8,9^.

The significant associations between MOR index scores and participants’ characteristics provide valuable insights into factors influencing women’s perceptions of respect during childbirth. Regional variations in childbirth experiences, as indicated by differences in MOR index scores based on the participants’ area of residence, may reflect disparities in access to maternity care services and cultural norms surrounding childbirth^14-16^. Similarly, the association between profession and MOR index scores underscores the influence of socio-economic factors and occupational backgrounds on women’s experiences of respectful maternity care^3,17^.

The sample’s demographics highlight a diverse representation, with a significant majority being of Greek nationality and residing in Attica. This is in line with existing literature suggesting that urban areas often attract a higher concentration of healthcare services and thus may influence women’s choices regarding childbirth location^18,19^. Additionally, the participants’ education level and marital status reflect a relatively well-educated and predominantly married population, consistent with findings from previous studies on maternal demographics^20^.

The high attendance rate of antenatal preparation courses with a midwife, underscores the importance of such programs in empowering women and preparing them for childbirth^21,22^. Furthermore, the involvement of midwives and doulas during home births, highlights the growing trend towards alternative birthing options and the role of supportive care providers in facilitating positive childbirth experiences^23,24^. Moreover**,** the findings regarding participants’ awareness of childbirth rights and laws reflect varying degrees of knowledge across different domains, echoing similar observations in other settings^25^.

Previous studies have emphasized the critical role of respectful maternity care in improving maternal health outcomes. Respectful care is linked to better health-seeking behaviors, positive birth experiences, and improved postpartum outcomes^5,6^. The validation of the MOR index in diverse cultural settings has shown promising results, demonstrating its adaptability and utility across different healthcare contexts^6^. For instance, studies in high-resource countries have highlighted the importance of measuring disrespect and abuse during childbirth to improve care quality^8,9^. The present study aligns with these findings, contributing to the global understanding of respectful maternity care.

### Strengths and limitations

The study’s strengths include its methodology which involves a retrospective online survey among women with at least one home childbirth experience in Greece and covers a broad period (2010–2023), allowing for the analysis of trends and changes in home birth practices and experiences over time. By focusing exclusively on women who have experienced home birth, the study provides in-depth insights into this particular childbirth option, contributing valuable information to a relatively under-researched area. The inclusion criteria ensure that participants have recent and relevant experience (within the last ten years), contributing to the study’s present relevance. Employing the MOR index to measure respectful care offers a standardized method to assess the quality-of-care women received during home births, contributing to the objectivity and comparability of the results. The confirmatory factor analysis and the use of Cronbach’s α reliability coefficient for questionnaire validation provide a robust statistical basis for evaluating the survey’s reliability and the structure of the data collected.

The study has, however, some limitations. The recruitment strategy, which relied on social media and online groups, might have led to a self-selection bias, as participants who are more active online or have stronger opinions about home birth may be overrepresented. The retrospective nature of the survey could introduce recall bias, with participants potentially misremembering details about their childbirth experience. Given the specific focus on women in Greece who have had home births, the findings may not be generalizable to women in other countries or to those who choose hospital births. Using an online self-administered questionnaire can lead to response bias, as participants might provide socially desirable answers or misinterpret questions without the opportunity for clarification. It is important to note that the study had a limited number of cases, which may affect the generalizability of the findings. Additionally, the analysis did not determine whether home births increased or decreased over the years.

## CONCLUSIONS

The validation of the Greek version of the Mothers on Respect (MOR) index may help promote the appropriate measurement of respectful maternity care and improve maternal health outcomes in Greece. Key findings highlight a high level of autonomy in decision-making, with many women feeling comfortable asking questions and declining care when necessary. Respect for personal and cultural preferences was also reported as high among participants. Most women experienced respectful treatment by healthcare professionals without feeling discriminated against based on race, ethnicity, or other factors. Open communication was emphasized, and participants could freely discuss their concerns and preferences. These results underscore the importance of personalized and respectful care in maternity services. Future research should explore the impact of these factors on maternal and infant health outcomes and develop targeted interventions to enhance respectful care practices across diverse settings. This study contributes to the broader global effort to ensure positive childbirth experiences for women worldwide and provides a reliable tool for assessing respectful maternity care in the Greek context.

## Supplementary Material



## Data Availability

The data supporting this research are available from the authors on reasonable request.
