# Supplementary material for: Validation of the Greek version of Mothers on Respect (MOR) index
Source: Eur J Midwifery. 2025 Jan 15;9:10.18332/ejm/196694. doi: 10.18332/ejm/196694 (PMC11734315; doi:10.18332/ejm/196694)
Supplement: Supplementary file 1 [file EJM-9-04-s1.pdf]

QUESTIONNAIRE

SECTION 1: Demographics

1. Age (Years):

2. Nationality

1. Greek

2. Other

3. Prefecture of residence

1. Attica

2. Thessaloniki

3. Evoia

4. Voiotia

5. Achaia

6. Other

4. Educational status

1. No education

2. Primary school

3. High school

4. Technical school

5. Vocational school for two years of training

6. Bachelor’s degree

7. Master’s degree

8. Doctorate

5. Marital status

1. Married

2. Single

3. Divorced

4. Widowed

5. Cohabitation agreement

6. Cohabitation

7. Other

6. Number of children:

SECTION 2: Data regarding preparation for home birth, data regarding income and profession before it and data on which professionals attended the home birth

7. In your FIRST home birth, did you attend antenatal preparation courses with a midwife?

1. Yes

2. No

3. I am a midwife

8. Your monthly household income during your FIRST home birth was:

1. 500-1000€
2. 1000-2000€
3. 2000-3000€
4. 3000-4000€
5. >4000€

9. What was your job when you had your FIRST home birth?

1. Private employee
2. Civil servant
3. Self-employed
4. Retired
5. Health care professional
6. Midwife
7. Unemployed
8. Householder
9. Other

10. Which professionals attended your FIRST home birth?

1. 2 midwives
2. 1 midwife
3. 1 midwife and 1 doula
4. 1 midwife and 1 gynecologist
5. 1 doula
6. Gynecologist
7. Pediatrician
8. Acupuncturist
9. Reflexologist

10. Osteopath
11. Unassisted (absence of health care professional)
12. Other

SECTION 3: Knowledge about childbirth rights and laws

11. In what extent do you know about:

	Not at all	Slightly	Moderately	Very well	Extremely well
**The rights of hospitalized patient?**					
**The current home birth laws?**					
**The children's rights?**					
**Your sexual and reproductive rights?**					

ΚΛΙΜΑΚΑ MOR: ΚΛΙΜΑΚΑ ΓΙΑ ΜΗΤΕΡΕΣ ΩΣ ΠΡΟΣ ΤΟ ΣΕΒΑΣΜΟ

ΟΔΗΓΙΕΣ

ΚΛΙΜΑΚΑ ΓΙΑ ΜΗΤΕΡΕΣ ΩΣ ΠΡΟΣ ΤΟ ΣΕΒΑΣΜΟ MOR

Η κλίμακα για Μητέρες ως προς το Σεβασμό MOR αναπτύχθηκε για να αξιολογήσει τη φύση της σχέσης ασθενούς – παρόχου υγείας και τον αντίκτυπό της στην αίσθηση της γυναίκας κατά τη φροντίδα που λαμβάνει στη διάρκεια της εγκυμοσύνης, της γέννας και συνολικά στη μητρότητα. Η κλίμακα MOR είναι ένας δείκτης ποιότητας και ασφάλειας προερχόμενος από την ίδια την γυναίκα, που μπορεί να εφαρμοστεί για την αξιολόγηση όλων των ειδών των σχέσεων παρόχων υγείας και ασθενούς και μπορεί επίσης να εφαρμοστεί και σε ατομοκεντρική φροντίδα (Vedam et al, 2017).

Η κλίμακα MOR αναπτύχθηκε μέσω μιας συμμετοχικής ερευνητικής διαδικασίας και έχει αναπτυχθεί και χορηγείται σε γυναίκες στον Καναδά και τις ΗΠΑ (βλ. το παρόν έγγραφο) και αποτελεί ένα αξιόπιστο και έγκυρο μέτρο για το σεβασμό στην φροντίδα της μητρότητας. Οι έγκυες ή οι λεχωΐδες μπορούν να χρησιμοποιήσουν την παρούσα κλίμακα των 14 στοιχείων, για να αξιολογήσουν το επίπεδο της άνεσής τους, τον αντίκτυπο στην προθυμία τους να κάνουν ερωτήσεις, ή/και τις αντιλήψεις για το ρατσισμό ή/και τις διακρίσεις κατά τη διάρκεια της περίθαλψής τους [6 διαφορετικές επιλογές για κάθε στοιχείο (βλ. παρακάτω)].

Τα πρώτα επτά στοιχεία της κλίμακας είχαν καλή απόδοση από μόνα τους και μπορούσαν να χρησιμοποιηθούν ως ξεχωριστή κλίμακα για τη μέτρηση της αίσθησης αυτονομίας και άνεσης ενός ατόμου, όταν αποδέχεται ή απορρίπτει επιλογές φροντίδας.

Για περισσότερες πληροφορίες σχετικά με την ανάπτυξη της κλίμακας MOR διαβάστε τη δημοσίευση του 2017 εδώ.

Αυτή η κλίμακα είναι δωρεάν για χρήση σε ακαδημαϊκά περιβάλλοντα, ωστόσο για οποιαδήποτε μελέτη που διεξάγεται από διαφημιστικό οργανισμό ή εάν η μελέτη υποστηρίζεται εν όλω ή εν μέρει από εμπορικό ή κυβερνητικό οργανισμό μπορεί να καταβληθεί οικονομικό τέλος. Αυτή η χρέωση εξαρτάται από διάφορους παράγοντες, για πληροφορίες επικοινωνήστε με το εργαστήριο Birth Place απευθείας: laura.beer@ubc.ca

zxereatlve �commons This work is licensed under the Creative Commons Auribution 4.0 International License. To view a copy of this license, visit hup://creativecommons.org/licenses/by/4.0/ or send a letter to Creative Commons, PO Box 1866, Mountain View, CA 94042, USA

Τυχόν κεφάλαια που προκύπτουν μέσω της συμφωνίας πνευματικών δικαιωμάτων χρησιμοποιούνται αποκλειστικά για την υποστήριξη του συνεχιζόμενου έργου του εργαστηρίου Birth Place.

Για ακαδημαϊκές σπουδές, δεν υπάρχει χρέωση, αλλά απαιτείται Συμφωνία Χρήστη για κάθε μελέτη. Σας παρακαλούμε κάντε κλικ εδώ για να συμπληρώσετε τη φόρμα αιτήματος λήψης.

Επικοινωνήστε μαζί μας για περισσότερες πληροφορίες σχετικά με τη χρήση: laura.beer@ubc.ca

Έχουμε επίσης αναπτύξει μια κλίμακα Μητρικής Αυτονομίας στη Λήψη Αποφάσεων (MADM) που μπορεί να χρησιμοποιηθεί παράλληλα με αυτή την κλίμακα ή από μόνη της. Η κλίμακα MADM είναι μια αξιόπιστη, έγκυρη και καθοδηγούμενη από τον ασθενή κλίμακα που μετρά την ικανότητα ενός ατόμου να ηγείται της λήψης αποφάσεων κατά τη διάρκεια της φροντίδας μητρότητας (Vedam et al, 2017).

Και οι δύο κλίμακες είναι επίσης διαθέσιμες για λήψη σε πολλές γλώσσες. Παρακαλώ επικοινωνήστε μαζί μας εάν αναζητάτε επικυρωμένες μεταφράσεις σε άλλες γλώσσες.

MOR ΠΑΡΑΠΟΜΠΗ:

Vedam, S., Stoll, K., Rubashkin, N., Martin, K., Miller-Vedam, Z., Hayes-Klein, H., & Jolicoeur, G. (2017). The Mothers on Respect (MOR) index: measuring quality, safety, and human rights in childbirth. SSM - Population Health, 3, 201-210. http://dx.doi.org/10.1016/j.ssmph.2017.01.005

zxereatlve �commons This work is licensed under the Creative Commons Auribution 4.0 International License. To view a copy of this license, visit hup://creativecommons.org/licenses/by/4.0/ or send a letter to Creative Commons, PO Box 1866, Mountain View, CA 94042, USA

MOR: ΚΛΙΜΑΚΑ ΓΙΑ ΜΗΤΕΡΕΣ ΩΣ ΠΡΟΣ ΤΟ ΣΕΒΑΣΜΟ

Παρακαλούμε, πείτε μας για τις συζητήσεις που είχατε με το γιατρό ή τη μαία σας σχετικά με τις επιλογές σας για τη φροντίδα σας στην εγκυμοσύνη και τη γέννα (για παράδειγμα: τον προγεννητικό έλεγχο, την έναρξη του τοκετού, τη χορήγηση ή μη των φαρμάκων, το πού θα γεννήσετε, τη φροντίδα του νεογνού, το αν θα κάνετε καισαρική τομή, κλπ.).

Οι απαντήσεις μου περιγράφουν τις συζητήσεις μου ή τις εμπειρίες μου με:

Τον οικογενειακό γιατρό

Τη μαία

Το Γυναικολόγο / Μαιευτήρα

Δεν ταιριάζουν με κάποιο συγκεκριμένο πρόσωπο, δεν είχα γιατρό ή μαία

**Α: Γενικά κατά τη λήψη αποφάσεων σχετικά με τη φροντίδα της εγκυμοσύνης μου ή του τοκετού μου:** (επίλεξε ή κύκλωσε μία απάντηση σε κάθε περίπτωση)						
	**Διαφωνώ έντονα**	**Διαφωνώ**	**Κάπως διαφωνώ**	**Κάπως Συμφωνώ**	**Συμφωνώ**	**Συμφωνώ απολύτως**
Ένιωθα άνετα κάνοντας ερωτήσεις	1	2	3	4	5	6
Ένιωθα άνετα απορρίπτοντας τη φροντίδα που μου πρόσφεραν	1	2	3	4	5	6
Ένιωθα άνετα αποδεχόμενη τη φροντίδα που μου πρόσφερε ο γιατρός ή η μαία μου	1	2	3	4	5	6
Ένιωθα ότι με πίεζε ο γιατρός ή η μαία μου να αποδεχτώ τη φροντίδα που μου πρότειναν	6	5	4	3	2	1
Επέλεξα εγώ η ίδια τις επιλογές στη φροντίδα που έλαβα	1	2	3	4	5	6
Οι προσωπικές επιλογές μου έγιναν σεβαστές	1	2	3	4	5	6
Οι πολιτισμικές μου προτιμήσεις έγιναν σεβαστές	1	2	3	4	5	6
**ΒΑΘΜΟΛΟΓΙΑ ΤΜΗΜΑΤΟΣ Α:**						
**Β: Κατά τη διάρκεια της εγκυμοσύνης μου ένιωσα ότι ο γιατρός ή η μαία μου δεν μου συμπεριφέρονταν σωστά λόγω:** (επίλεξε ή κύκλωσε μία απάντηση σε κάθε περίπτωση)						
	**Διαφωνώ έντονα**	**Διαφωνώ**	**Κάπως διαφωνώ**	**Κάπως Συμφωνώ**	**Συμφωνώ**	**Συμφωνώ απολύτως**
Της φυλής, της εθνικότητάς μου, του πολιτισμικού μου υπόβαθρου ή της γλώσσας μου*	6	5	4	3	2	1
Του σεξουαλικού μου προσανατολισμού ή/και της ταυτότητας του φύλου μου*	6	5	4	3	2	1
Του είδους ασφάλισης υγείας μου ή της έλλειψης ασφάλισης*	6	5	4	3	2	1
Της διαφοράς απόψεων με τους φροντιστές μου για τη σωστή φροντίδα της δική μου και του μωρού μου*	6	5	4	3	2	1
**ΒΑΘΜΟΛΟΓΙΑ ΤΜΗΜΑΤΟΣ Β:**				**ΣΥΝΟΛΙΚΗ ΒΑΘΜΟΛΟΓΙΑ ΤΜΗΜΑΤΟΣ Α+Β:**		
**Γ: Κατά τη διάρκεια της εγκυμοσύνης συγκρατιόμουν από το να κάνω ερωτήσεις ή να συζητώ προβληματισμούς γιατί:** (επίλεξε ή κύκλωσε μία απάντηση σε κάθε περίπτωση)						
	**Διαφωνώ έντονα**	**Διαφωνώ**	**Κάπως διαφωνώ**	**Κάπως Συμφωνώ**	**Συμφωνώ**	**Συμφωνώ απολύτως**
Ο γιατρός ή η μαία μου φαίνονταν να βιάζονται*	6	5	4	3	2	1
Η φροντίδα που εγώ ήθελα ήταν διαφορετική από αυτή που πρότεινε ο γιατρός ή η μαία	6	5	4	3	2	1
Πίστευα ότι ο γιατρός ή μαία θα νόμιζαν ότι είμαι περίεργη	6	5	4	3	2	1
**ΒΑΘΜΟΛΟΓΙΑ ΤΜΗΜΑΤΟΣ Γ:**				**ΣΥΝΟΛΙΚΗ ΒΑΘΜΟΛΟΓΙΑ Α+Β+Γ:**		

zxereatlve �commons This work is licensed under the Creative Commons Auribution 4.0 International License. To view a copy of this license, visit hup://creativecommons.org/licenses/by/4.0/ or send a letter to Creative Commons, PO Box 1866, Mountain View, CA 94042, USA

Πίνακας Βαθμολογίας	
Εισάγετε τη συνολική βαθμολογία του τμήματος Α	
Εισάγετε τη συνολική βαθμολογία του τμήματος Β	
Εισάγετε τη συνολική βαθμολογία του τμήματος Γ	
Α + Β + Γ = ΣΥΝΟΛΙΚΗ ΒΑΘΜΟΛΟΓΙΑ	

Η βαθμολογία κυμαίνεται μεταξύ 14-84, με το υψηλότερο σκορ να δείχνει φροντίδα με σεβασμό.

Επίπεδο Σεβασμού, όπως βιώθηκε από τη γυναίκα (ανά τεταρτημόρια)	
**Συνολική Βαθμολογία**	**Επίπεδο Σεβασμού**
14-31	Πολύ Λίγος Σεβασμός
32-49	Λίγος Σεβασμός
50-66	Μέτριος Σεβασμός
67-84	Υψηλός Σεβασμός

*Αντικείμενα με αντίστροφη βαθμολογία

© 2024 Serpetini E. et al.

zxereatlve �commons This work is licensed under the Creative Commons Auribution 4.0 International License. To view a copy of this license, visit hup://creativecommons.org/licenses/by/4.0/ or send a letter to Creative Commons, PO Box 1866, Mountain View, CA 94042, USA
